# Preliminary observations of muscle fibre cross sectional area of flexor digitorum brevis in cadaver feet with and without claw toes

**DOI:** 10.1186/1757-1146-3-32

**Published:** 2010-12-22

**Authors:** Jackie Locke, Stuart A Baird, Jamie Frankis

**Affiliations:** 1 Department of Podiatric Medicine and Surgery, Glasgow Caledonian University, Cowcaddens Road, Glasgow, Scotland, G4 OBA, United Kingdom

## Abstract

**Background:**

In order to facilitate normal gait, toes require to be in a rectus position during the propulsive phase. This requires a correct balance and sequence of activity of the intrinsic musculature of the feet. Alteration of this balance and sequence may lead to the development of claw toes. Atrophy of the lumbricals occurs in the development of claw toes, but it is not known if changes occur in any other intrinsic muscles, including flexor digitorum brevis. This study set out to investigate whether hypertrophic changes were evident in flexor digitorum brevis in feet with claw toes.

**Methods:**

Four cadaver feet were investigated, two with rectus toes and two with claw toes. Flexor digitorum brevis was removed from each, and seven anatomically significant tissue sections from each muscle were routinely processed, cut and stained. One hundred and sixty muscle fibre cross sectional areas were measured from each section.

**Results:**

The mean age of the donors was 81.5 years, and three of the four were female. Results showed that the cross sectional area of fibres from feet with claw toes was 417 μg^2 ^significantly greater (p < 0.01) than the cross sectional area of fibres from feet with rectus toes, which was 263 μg^2^.

**Conclusions:**

Although this study has several limitations, preliminary observations reveal that flexor digitorum brevis muscle fibre cross sectional area is significantly reduced in feet with claw toes. This would indicate a relationship between muscle fibre atrophy of flexor digitorum brevis and clawing of the lesser toes.

## Background

Toes have the primary function of increasing the total weight bearing area of the forefoot during the stance phase of gait, dispersing the loads under the metatarsal heads [1]. If optimal propulsion is to occur, the toes should be parallel with the ground at approximately 20° of metatarsophalangeal (MTP) joint dorsiflexion in relation to the metatarsal shaft [2]. The toes must also be stable and function in a rectus position as a rigid beam [3]. During the propulsive phase of gait, the stable digit becomes the point about which muscle activity occurs [3]. Correct digital function is a balance between the intrinsic and extrinsic muscularae acting on the digits during the gait cycle. If the rectus position is to be maintained in order to facilitate effective propulsion, a sequence of muscle contractions must occur. When smooth co-ordination of the muscle groups is compromised, the dominant group gains a mechanical advantage, altering the digital position during propulsion [3,4]. This, coupled with Davis's Law, leads to contractures and digital deformities [3].

Claw toes are part of a group of lesser toe deformities, which also include mallet and hammer toes. They are best defined as a sagittal plane deformity, where there is dorsiflexion at the MTP joint, and flexion of the intermediate and distal interphalangeal (IP) joints [1,5,6]. There are numerous aetiological factors associated with the development of claw toes including muscular spasm resulting from an upper motor neurone lesion, rheumatoid arthritis, biomechanical abnormalities, inappropriate footwear, peripheral neuropathy, neuromuscular disorders and myoneural ischemia from compartment syndromes of the foot and calf [1,7]. Whilst a wide spectrum of aetiological factors is evident, most authors are agreed that they all result in pathomechanical changes that cause muscular imbalance, bringing about alterations in digital position.

It is accepted that clawing of the toes is associated with atrophy of the lumbrical muscles, [3,4,8] however no quantifiable data has been produced as evidence to support this. Root et al. [4] suggest that the lumbricals are stance phase muscles which act in conjunction with flexor digitorum longus (FDL). Lumbrical function is to extend the intermediate and distal IP joints of the lesser toes [4] and assist in stabilizing the proximal phalanx of the lesser toes by plantarflexing against ground reaction force [4]. This action maintains the digits in a rectus position during gait, but atrophy would permit overpull of the flexors to extend the MTP joints against ground reaction force, and flex the intermediate and distal IP joints to create a claw toe deformity. Greene and Brekke [3] dispute this stance phase theory, stating that lumbricals are primarily swing phase muscles, which are activated to produce a flexion force at the MTP joints prior to the activation of extensor digitorum longus and brevis, thus creating digital stability prior to the toes commencing ground contact.

Atrophy or paralysis of the lumbricals [9] disrupts both the sequence and balance of muscle activity during gait, resulting in a weakened or unopposed action of the extensors during swing. As a consequence of the reduced flexion action of the lumbricals at the MTP joints, there is extension of the MTP joints during the swing phase, resulting in the digits being in an unstable position prior to ground contact. Coupled with the actions of FDL and flexor digitorum brevis (FDB) through the propulsive phase, flexion of the proximal and distal IP joints occurs, with all joints in the claw toe position [3,8,9]. Whether stance or swing phase muscles, or both, lumbrical atrophy or paralysis would result in clawing of the lesser toes. However, the role of other muscles requires investigation to gain a greater understanding of this deformity. If, as Greene and Brekke [3] suggest, there is a mechanical overpull of FDB and FDL, they may display evidence of hypertrophic changes.

FDB is a fusiform muscle originating from the medial calcaneal process and the plantar aponeurosis. At its distal aspect, it divides into four, giving rise to four tendons, each of which insert into the lateral four toes on the plantar surface of the intermediate phalanx [10]. Because of the site of the tendonous insertions, any implication of FDB in the development of claw toes can only relate to changes at the MTP joints and proximal IP joints, but not the distal IP joints. The open chain action of FDB is to flex the MTP joints and proximal IPJ's, [10], but its function during gait is somewhat less clear. Amongst the various functions described by Root et al. [4], two relate specifically to digital stability. FDB (i) assists flexor digitorum longus to maintain stability of the lesser digits against the ground in propulsion, and (ii) stabilises the intermediate phalanx of each toe posterially against the proximal phalanx, and the proximal phalanx against its respective metatarsal head. These functions would maintain the digits in a rectus position. Root et al. [4] also describe FDB as a stance phase muscle, a theory concurrent with the findings of Greene and Brekke [3], Hughes [2] and Perry [11]. The timing of its activity during the stance phase of gait is disputed, with some authors quoting as little as 33%, and others up to 75% [2,4,11]. However, all are agreed that FDB is active from heel lift through toe off, to maintain the digits in a rectus position, thus obtaining the necessary stability during the propulsive phase of gait. According to Greene and Brekke [3], FDB also works in conjunction with FDL during normal gait to ensure the toe remains flat against the ground during stance, to create the rigid beam required for effective propulsion. An excessive unbalanced action, or a pull which is unopposed by correct lumbrical function would lead to extension of the MTP joints against ground reaction force, and flexion of the proximal IP joints. It is reasonable to conclude that FDB should be investigated in relation to the development of claw toes.

The ability to produce force and movement is the most basic property of skeletal muscle [12,13]. It does so by contracting either isometrically, where tension is produced against force without the muscle shortening, or isotonically where the muscle contracts to produce movement [12,13]. There are three main influences on muscle contraction, namely fibre type, length and diameter [14,15]. To investigate fibre type and length was outwith the scope of this study. However the importance of fibre length was recognised as individual muscle fibres do not extend the full length of the muscle itself, but are arranged in overlapping bundles [16]. Investigations by Loeb et al. [17], and Ounjian et al. [18] revealed that many muscle fibres originate and terminate within the muscle belly, attaching to the connective tissue matrix, which itself has an important role in fibre to tendon tension. Given this, several sites of anatomical significance were selected for measuring. Muscle fibre diameter was not measured directly, as fibres are not perfectly round. However, cross sectional fibre area, which is directly related to fibre diameter, was measured by computer based image analysis. This method has been shown to have both intra and inter tester validity and reliability [19]. Any alterations in mechanical usage or innervation to the muscle will result in either atrophic or hypertrophic changes to the muscle, which is manifest by a decrease or increase respectively in the size of the individual muscle fibre [20]. The aim of the study was to test the hypothesis that the muscle fibre cross sectional area of FDB was greater in feet with claw toes than in feet with rectus toes.

## Methods

For the purpose of this study, fifteen feet were made available by Glasgow University Department of Human Anatomy. They were then sub divided into two groups, feet with rectus toes, and feet with claw toes. Two feet from each sub group were then randomly selected. The inclusion criterion for rectus toes was clinical observation of all lesser toes in a rectus position. The inclusion criterion for claw toes was clinical observation of all lesser toes in the claw position as defined by Merriman and Tollafield [6]. The exclusion criteria for rectus toes was any single toe with another lesser toe deformity such as hammer or mallet toe, or any indication of trauma or surgical procedure to the foot. The exclusion criteria for claw toes were any lesser toe in a rectus position, or any indication of trauma or surgical procedure to the foot.

**Figure 1:**
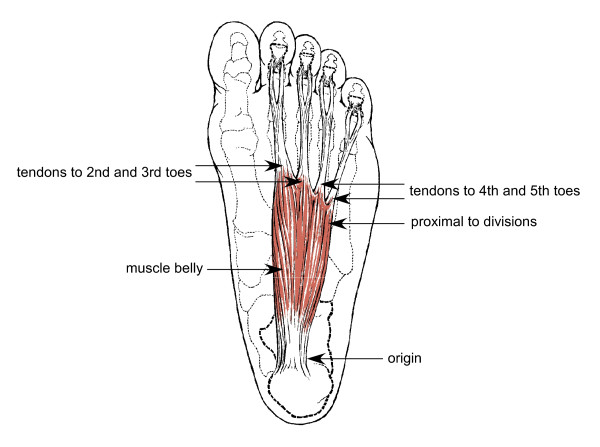
**Diagramatical illustration of anatomical sites of muscle tissue sections taken from flexor digitorum brevis**.

The flexor digitorum brevis of each foot was removed by minute dissection as described by Romanes [10]. A 3 mm transverse section was then isolated from seven sites within the muscle (Figure 1).

Each section was routinely decalcified for 7 days in Ethylenediaminetracetic acid (EDTA), processed via the Histokinette 2000 (Reichert-Jung, Germany) and vacuum embedded in wax at 58°C, cooled for a minimum of 2 hours to solidify, prior to being cut to 7 μm. They were then routinely mounted and stained using Haematoxylin and Eosin (H&E). Visual image analysis was conducted using an Olympus BH2 RFCA (Olympus, London) photo microscope, in conjunction with Soft Image Analysis^® ^and Viewfinder Lite^® ^Version 1.0 (Pixera Corporation 1998 - 2000). Using this software, tissue samples from each section were divided into 4 quadrants. Within each quadrant a blood vessel was located, and the cross sectional areas of the surrounding 40 muscle fibres measured at × 40,000 magnification. This data was recorded directly to Microsoft^® ^Excel 1997 (Microsoft^® ^Corporation, USA) and copied to SSPS 12.0^© ^2002 SPSS Inc.for statistical analysis.

## Results

**Figure 2:**
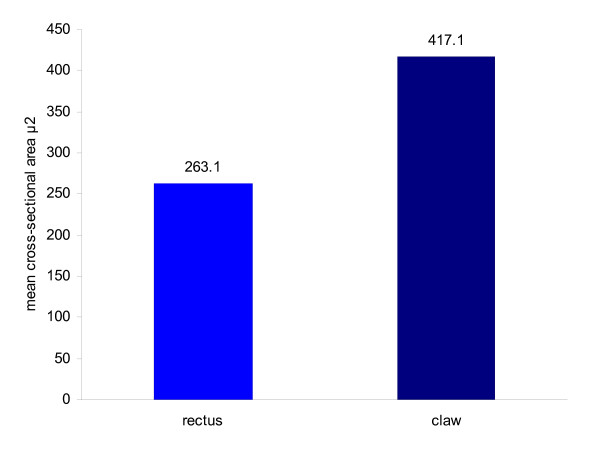
**Comparison of mean cross sectional areas in feet with rectus toes and feet with claw toes**.

FDB was dissected from four cadaver feet. Foot 2 had no tendon attached to the 5^th ^toe. Table 1 gives details of age, gender, mean cross sectional area, standard deviation and range for each foot. Figure 2 illustrates that the mean cross sectional area is greater in feet with claw toes than feet with rectus toes.

**Table 1:** Characteristics of feet included in the sample

Foot	Foot 1	Foot 2	Foot 3	Foot 4
Type	**Rectus**	**Claw**	**Rectus**	**Claw**

Age	**71**	**88**	**83**	**84**

Gender	**Male**	**Female**	**Female**	**Female**

Mean in μ^2^	**259.74**	**341.70**	**239.89**	**430.44**
(Standard Deviation)	**(123.08)**	**(199.97)**	**(106.20)**	**(222.64)**

Range in μ^2^	**63.86 - 1212.10**	**80.41- 1525.87**	**61.75- 912.79**	**89.87 1403.41**

Independent sample t-tests showed;

(i) Mean cross sectional area associated with clawed toes (417 μ^2^) was significantly greater than the cross sectional area associated with rectus toes (263 μ^2^) (p = < 0.01), with a difference of the means = 154 μ^2^.

(ii) A significant difference in the mean cross sectional area (260 μ^2 ^and 240 μ^2^) of the two feet with rectus toes (p < 0.01) with a difference of the means = 20 μ^2^.

(iii) A significant difference in the mean cross sectional area (342 μ^2 ^and 493 μ^2^) of the two feet with claw toes (p < 0.01) with a difference of the means 151 μ^2^.

**Figure 3:**
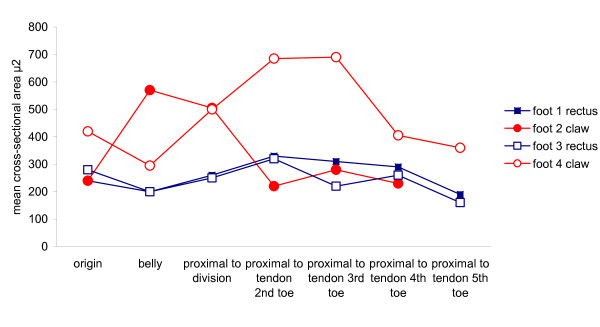
**Comparison of muscle fibre cross sectional areas within each foot**.

The two rectus feet displayed a similar pattern of muscle fibre cross sectional area at each anatomical site examined. Foot 4 (claw) had a greater cross sectional area than both rectus feet at each anatomical site, whereas Foot 2 (claw) was only greater than both rectus feet at 2 sites. This made a conclusion problematic and can be viewed diagrammatically in Figure 3. In the light of this statistical analysis, and due to alterations in ageing muscle fibres, the cross sectional area from the 83 year old female rectus foot was compared with the 84 year old female claw foot at each anatomical site. A two way ANOVA which investigated person and anatomical site revealed that the cross sectional area of the claw foot was significantly greater (p = < 0.01), than the cross sectional area of the rectus foot at all corresponding anatomical sites (Table 2).

**Table 2:** Results of two-way analysis of variance comparing muscle cross sectional area at different sites in feet with and without claw toes.

				95% Confidence Interval		
					
Anatomical site	Foot number (type)	Mean	Std. error	Lower bound	Upper bound	ANOVA p-value
Origin	3 (rectus)	276.57	10.99	255.02	298.12	<0.01
	4 (claw)	424.30	10.99	402.75	445.86	<0.01

Belly	3 (rectus)	201.62	10.99	180.06	223.17	<0.01
	4 (claw)	285.65	10.99	264.10	307.21	<0.01

Prox div	3 (rectus)	234.86	10.99	213.31	256.42	<0.01
	4 (claw)	478.52	10.99	456.70	500.07	<0.01

Tendon 2^nd^	3 (rectus)	325.06	10.99	303.51	346.61	<0.01
	4 (claw)	676.90	10.99	655.35	698.47	<0.01

Tendon 3^rd^	3 (rectus)	228.91	10.99	207.36	250.46	<0.01
	4 (claw)	679.68	10.99	658.13	701.24	<0.01

Tendon 4^th^	3 (rectus)	256.76	10.99	235.20	278.31	<0.01
	4 (claw)	410.21	10.99	388.66	431.77	<0.01

Tendon 5th	3 (rectus)	155.43	10.99	133.87	176.98	<0.01
	4 (claw)	357.70	10.99	336.15	379.25	<0.01

## Discussion

The findings of this study appear to confirm the hypothesis that mean cross sectional area is greater in FDB associated with claw toes than rectus toes. This however is a simplistic analysis, and several issues should be considered to ensure that this finding is viewed within the appropriate context. Further investigation revealed there was a significant difference in the mean cross sectional area of the two rectus feet and the two claw feet. The difference between the means of the two claw feet was 151 μ^2 ^, whilst the difference of the means between claw and rectus feet was only 3 μ^2 ^greater at 154 μ^2 ^. When the mean cross sectional area for each of the anatomical sites of the four feet were contrasted, as displayed in Figure 3 it was noted that not all anatomical sites of the claw feet were greater than the same anatomical site in the rectus feet.

The two rectus feet followed a similar pattern of cross sectional area at each anatomical site within the muscle, whereas the two claw feet did not. Foot 4 was greater at each anatomical site than both rectus feet, but apart from the sites of Belly and Proximal to Division of Foot 2, the measurements from this claw foot were similar in size and pattern to the two rectus feet. It is therefore not entirely accurate to conclude that the cross sectional area is greater in claw feet than rectus feet.

One of the major influences on these results, is the age of the sample. The youngest was 71 years, with the other three aged 83, 84 and 88 years. Inokuci [21] concluded that muscle tissue is marked by an increase in fat and connective tissue in the elderly, especially those in advance of 80 years. During dissection, it was noted in the 88 year old that visually, there appeared to be a greater amount of fat surrounding and impregnating the muscle. Additionally, under microscopic conditions, an increase in the endomysial spaces was visually apparent. Hooper [22] recorded that one characteristic of ageing muscle is an increased variation in fibre size due to atrophy and compensatory hypertrophy. Table 1 displays the maximum and minimum measurements, and indicates an exceptionally large range of fibre sizes, giving credence to this theory. There appear to be two theories as to why there is fibre atrophy with compensatory hypertrophic changes. Firstly, as age increases above 60 years, neurogenic alterations occur, resulting in cycles of denervation and reinnervation of motor neurons. With each cycle, some fibres are permanently denervated, resulting in atrophy and are eventually replaced by connective tissue [23]. Secondly, a reduction in the effectiveness of the peripheral arterial supply due to arteriosclerosis is a physiological process that occurs with advancing age [24]. This reduces the healing properties in any muscle fibres which are injured, even during moderate physical activity, resulting in eventual muscle loss. As a consequence of either occurrence, a reduced number of fibres would be required to maintain the same activity, resulting in hypertrophic changes. Grimby and Saltin [25] suggest that neurogenic changes affecting muscle fibres, are directly related to the length of the peripheral nerve, thereby implying there is a likely risk of these changes affecting muscles of the foot. Atrophic changes will also arise from muscle disuse [13], due to reduction in ambulation as age increases. In the light of age related changes to muscle tissue, the 83 year old rectus and 84 year old claw (both female) feet were compared. A two-way ANOVA confirmed a significant difference between claw and rectus toes, with the cross sectional area of all corresponding anatomical sites greater in the foot with claw toes (Table 2).

It would also be prudent to observe five other limitations associated with this study. Firstly, the study was carried out on muscle tissue from cadavers treated with a Formaldehyde based embalming fluid as per Glasgow University embalming protocols. This may result in the tissues becoming hard, rigid and often difficult to dissect [26]. MacBride [27] also suggested that studies using embalmed cadavers were often avoided because the fixation process was poor, making tissues less suitable for histological examination. In examining the effects of various fixatives on bovine muscle, Stickland [28] observed that Formaldehyde based solutions were amongst those most likely to cause shrinkage to the muscle fibre. However, it should also be considered that the effects of shrinkage on skeletal muscle of cadaveric fixation was marked when muscle tissue was fixed in isolation from the skeleton, but not when fixed in situ on the skeleton [29]. Apart from the effects of the embalming solution, the subsequent stages of dehydrating and clearing have also been implicated in muscle fibre shrinkage. It has been concluded by Stickland [28] that these processes cause shrinkage to a greater degree than fixation. Histological processing can also cause fragmentation of tissue [30], and this was evident in some of the tissues under microscopic examination, occasionally making measurement difficult. In addition to the effects of fixation, dehydrating and clearing, muscle fibres taken from cadavers differ architecturally [31] from *in vivo *muscle. Living muscle fibres are either in an extreme of relaxation or contraction, whereas cadaveric muscle is in a state between relaxation and contraction [31]. This is thought to be a result of fixation occurring whilst the muscle fibres are in the partially contracted state seen in rigor mortis. Due to the alterations of fixation, histological processing and rigor mortis, results of cadaveric studies cannot reflect exactly what would be found in a living specimen. One final problem in conducting this cadaveric study was that no medical history was available that may have indicated an aetiological influence on fibre atrophy, hypertrophy or development of claw toes.

The second major limitation is that this investigation has only looked at the muscle fibre cross sectional area, but has not accounted for any differences associated with the other two components of muscle contraction, namely, fibre length and fibre type. Although fibre length was not investigated, observation of Figure 3 demonstrates that the mean cross sectional area varies at each of the various sections along the muscle. It is not known whether the fibre lengths or their origins and insertions to connective tissue varies between claw and rectus toes. Length of fibres from FDB would be observations in the sagittal plane, the same plane on which claw toe deformity occurs. Many biomechanical abnormalities associated with the development of claw toes are also associated with elongation of the medial longitudinal arch of the foot [32]. In such conditions, chronic stretching of muscle fibres could occur in FDB. As a result the fibre may increase in length, causing the muscle velocity, excursion and ability to generate force to increase [14]. This would be consistent with the theory that there is excessive pull of FDB associated with claw toes.

There was no possible means of making any observations relating to fibre type during this study, as H&E does not reveal any difference. Serrano et al. [33] illustrated not only the potential of muscle fibre to change fibre type, but the existence of "hybrid fibres" which can quickly undergo transitions from one fibre type to another, in response to changes of muscle activity. Fibre types have been seen to change in response to force, duration and velocity of muscle activity, all of which are relevant within the context of gait. It would be of extreme interest to note if there is a difference in proportion of fibre type between claw and rectus toes, and if the hypertrophic changes affect one fibre type more than another. Any findings from such a study would give great indication as to whether FDB is involved in active gait or postural stability.

The third major limitation is that this was a morphological study, which has investigated a functional pathology. In order to fully understand muscle function, especially within the foot, muscle activity requires to be studied during gait. The options for obtaining quantitative data from muscle activity from the foot are limited due to both the restrictions of the instrumentation and the anatomical positioning of foot muscles. EMG studies have been used, but possible limitations have already been documented. Magnetic resonance imaging has been extremely successful in the study of cadaveric muscle, but less successful when used for *in vivo *muscle activity [34]. Ultrasonographic studies have been documented as the method to provide a better understanding of the dynamic nature of skeletal muscle, and could be used to elucidate the biomechanics of muscle contraction [31].

Fourthly, it should be recognised that this study has only made observations regarding FDB in isolation from other muscles that may be implicated in the development of claw toes. In order to gain a true understanding of atrophic and hypertrophic changes, a comprehensive study of all muscles attaching to the lesser toes is required. This would facilitate a comparison not only of individual muscles and their differences in claw and rectus toes, but how muscles of the same foot compare between the two conditions.

A fifth point to note is that the sample used was small, with only four feet being analysed. Although a larger sample would give a clearer picture, conclusions have been drawn from previous cadaver studies using a similar sample size [31,34].

In order to gain more data, further studies in this field are required and should endeavour to select a larger sample with similar specimen ages to obtain a more meaningful comparison. It is also necessary to investigate all muscles which are potentially involved in the development of claw toes. To advance the understanding of muscle differences between claw and rectus toes at a morphological level, the investigation of fibre type would be advantageous. A high proportion of Type IIA and IIB fibres would indicate an active functional role in gait, as they produce high power output, but a high proportion of Type I fibres would indicate a link to postural stability. It is possible there may be a difference in proportion of fibre types between muscles associated with rectus toes, and those with claw toes. The study of fibre type using sigma antibodies to fast myosin is required.

## Conclusion

This research was undertaken to investigate whether there were hypertrophic changes associated with flexor digitorum brevis in the development of claw toes. The study was limited by variables such as small sample size, alterations to tissue associated with embalming and histological processing, only being able to investigate one influence of muscle contraction and lack of cadaver medical history. However, despite these limitations, quantifiable data has been produced in an area of anatomy that currently has received little or no investigation

## Competing interests

The authors declare that they have no competing interests.

## Authors' contributions

JL participated in the study design, carried out the dissection, histology, data collection, assisted with statistics and drafted the manuscript. SB conceived the study, and participated in its design and coordination and helped to draft the manuscript. JF directed and supervised the statistical analysis and interpretation of the study results, and commented on the drafts of the manuscript. All authors read and approved the final draft.

## Authors' Information

JL is a Lecturer in Department of Podiatric Medicine and Surgery, SB Head of Department of Podiatric Medicine and Surgery, and JF a Senior Lecturer in Research Methods within the School of Health. JL is also an Associate Teacher of Podiatry, Southern General Hospital, Glasgow, Scotland, United Kingdom, G51 4TF.
